# Proteomics of tumor and serum samples from isocitrate dehydrogenase‐wildtype glioblastoma patients: is the detoxification of reactive oxygen species associated with shorter survival?

**DOI:** 10.1002/1878-0261.13668

**Published:** 2024-05-27

**Authors:** Anne Clavreul, Catherine Guette, Hamza Lasla, Audrey Rousseau, Odile Blanchet, Cécile Henry, Alice Boissard, Mathilde Cherel, Pascal Jézéquel, François Guillonneau, Philippe Menei, Jean‐Michel Lemée

**Affiliations:** ^1^ Département de Neurochirurgie CHU d'Angers France; ^2^ Inserm UMR 1307, CNRS UMR 6075 Université de Nantes, CRCI2NA, Université d'Angers France; ^3^ PROT'ICO – Plateforme Oncoprotéomique Institut de Cancérologie de l'Ouest (ICO) Angers France; ^4^ Omics Data Science Unit Institut de Cancérologie de l'Ouest (ICO) Nantes France; ^5^ SIRIC ILIAD, Institut de Recherche en Santé, Université de Nantes France; ^6^ Département de Pathologie CHU d'Angers France; ^7^ Centre de Ressources Biologiques, BB‐0033‐00038 CHU d'Angers France; ^8^ Département de Biologie Médicale Centre Eugène Marquis, Unicancer Rennes France

**Keywords:** glioblastoma, IDH‐wildtype, metabolism, prognosis, proteomics, reactive oxygen species

## Abstract

Proteomics has been little used for the identification of novel prognostic and/or therapeutic markers in isocitrate dehydrogenase (IDH)‐wildtype glioblastoma (GB). In this study, we analyzed 50 tumor and 30 serum samples from short‐ and long‐term survivors of IDH‐wildtype GB (STS and LTS, respectively) by data‐independent acquisition mass spectrometry (DIA‐MS)‐based proteomics, with the aim of identifying such markers. DIA‐MS identified 5422 and 826 normalized proteins in tumor and serum samples, respectively, with only three tumor proteins and 26 serum proteins displaying significant differential expression between the STS and LTS groups. These dysregulated proteins were principally associated with the detoxification of reactive oxygen species (ROS). In particular, GB patients in the STS group had high serum levels of malate dehydrogenase 1 (MDH1) and ribonuclease inhibitor 1 (RNH1) and low tumor levels of fatty acid‐binding protein 7 (FABP7), which may have enabled them to maintain low ROS levels, counteracting the effects of the first‐line treatment with radiotherapy plus concomitant and adjuvant temozolomide. A blood score built on the levels of MDH1 and RNH1 expression was found to be an independent prognostic factor for survival based on the serum proteome data for a cohort of 96 IDH‐wildtype GB patients. This study highlights the utility of circulating MDH1 and RNH1 biomarkers for determining the prognosis of patients with IDH‐wildtype GB. Furthermore, the pathways driven by these biomarkers, and the tumor FABP7 pathway, may constitute promising therapeutic targets for blocking ROS detoxification to overcome resistance to chemoradiotherapy in potential GB STS.

## Graphical abstract

We used DIA‐MS‐based proteomics to detect changes in protein abundance in tumor and serum samples from short‐term (STS) and long‐term (LTS) glioblastoma survivors. The dysregulated proteins were principally associated with the detoxification of reactive oxygen species (ROS). High serum levels of MDH1 and RNH1 and low tumor levels of FABP7 may enable STS to maintain low ROS levels, counteracting the effects of chemoradiotherapy.

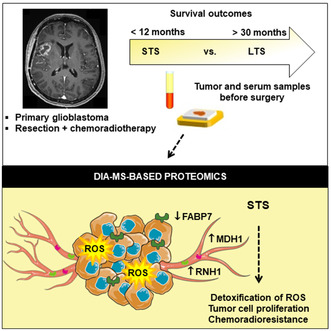

**Abbreviations**
AA: arachidonic acid
AUC: area under the curve
BBB: blood–brain barrier
BH: Benjamini–Hochberg
BRIF: bioresource research impact factor
CAFs: cancer‐associated fibroblasts
DHA: docosahexaenoic acid
DIA‐MS: data‐independent acquisition mass spectrometry
DMNC: density of maximum neighborhood component
EOR: extent of resection
FFPE: formalin‐fixed paraffin‐embedded
GASCs: glioma‐associated stromal cells
GB: glioblastoma
GTR: gross total resection (100%)
IDH: isocitrate dehydrogenase
IHC: immunohistochemistry
KPS: Karnofsky performance score
LTS: long‐term survivors
MCC: maximal clique centrality
MGMT: O(6)‐methylguanine methyltransferase
MIC: minimizing approximated information criteria
OS: overall survival
PCA: principal component analysis
PDACs: pancreatic ductal adenocarcinoma cells
PFS: progression‐free survival
PR: partial resection (< 90%)
ROC: receiver operating characteristic
ROS: reactive oxygen species
SAM: significance analysis of microarray
STR: subtotal resection (≥ 90%)
STS: short‐term survivors
TMZ: temozolomide

## 1 Introduction

Glioblastomas (GB) are the most common and aggressive primary tumors of the central nervous system. Before 2005, the standard of care was limited to surgical resection and radiation therapy. After 2005, surgical resection and the Stupp regimen [1, 2], which combines radiation therapy with concurrent and adjuvant chemotherapy with temozolomide (TMZ), became the standard of care in patients younger than 70 years. However, the prognosis of patients remains poor, with a median survival of about 15 months. An interesting exception to this rule is the small fraction of patients (< 15%), known as long‐term survivors (LTS), who survive beyond 36 months [3]. Survival seems to be more strongly associated with response to the standard first‐line treatment than with response to second‐line treatments, for which there is no broadly consensual approach [4]. Various clinical features related to longer survival have been identified, including younger age at presentation, high preoperative Karnofsky performance score (KPS), lack of tumor proximity to the subventricular zone, and more extensive surgical resection [5]. Molecular analyses have suggested that O6‐methylguanine DNA methyltransferase (*MGMT*) promoter methylation is a potential predictive marker for response to TMZ, and isocitrate dehydrogenase (IDH) mutations have been identified as markers of a favorable prognosis [5]. However, GB carrying IDH mutations have gene methylation and expression profiles different from those of IDH‐wildtype GB, as well as a different prognosis and response to treatment. This observation prompted the World Health Organization (WHO) to consider IDH‐mutated GB as IDH‐mutated grade 4 astrocytomas in its updated classification, to improve their distinction from IDH‐wildtype GB [6, 7].

Several genomic and transcriptomic data analyses comparing GB tissue samples from short‐term survivors (STS) and LTS have been performed to search for survival‐associated variants [8, 9, 10, 11, 12, 13, 14, 15, 16, 17, 18]. Novel biomarkers and potential actionable therapeutic targets have been proposed for GB management, but none have been integrated into clinical practice for prognostic assessment in patients with IDH‐wildtype GB. Yanovich‐Arad et al. [19] recently reported that the proteomic profiles of primary IDH‐wildtype GB were associated with tumor subtypes different from the established transcriptomic subtypes and more robustly associated with survival. These findings highlight the potential utility of proteomics approaches as a complementary means of identifying the molecular characteristics of LTS in the context of IDH‐wildtype GB. However, proteomics has been little used in the identification of prognostic markers for GB to date. Stetson et al. [20] have conducted a LC–MS/MS‐based proteomics analysis on tumor tissues from 27 GB patients: 13 STS (≤ 10 months) and 14 LTS (≥ 18 months). They found that proteins involved in axon guidance were upregulated in STS relative to LTS, whereas proteins involved in p53 signaling were upregulated in LTS. Several other studies have analyzed the serum or plasma proteomes of GB patients, comparing the results obtained with those for healthy subjects, but no proteomics study has ever compared blood samples from STS and LTS for GB [21, 22, 23, 24, 25, 26, 27, 28, 29].

Here, we assess the ability of data‐independent acquisition mass spectrometry (DIA‐MS) to detect differences in protein abundance in tumor and serum samples from STS and LTS with IDH‐wildtype GB. DIA‐MS is increasingly used for the quantification of proteins in clinical samples [30, 31, 32]. This proteomics approach is based on a full scan of all precursors, which are subsequently isolated and fragmented within a defined mass‐to‐charge (*m/z*) window until the entire *m/z* range of the initial full scan has been covered. DIA‐MS has been shown to be highly reproducible and precise for protein quantification. Proteins differing in abundance between STS and LTS could be useful as prognostic biomarkers and/or potential therapeutic targets in patients with IDH‐wildtype GB.

## 2 Materials and methods

### 2.1 Experimental design and statistical rationale

This study included patients newly diagnosed with IDH‐wildtype GB between January 2012 and December 2018 from the French GB biobank (FGB) [33]. The following inclusion criteria were used: (a) patient aged ≥ 18 years, (b) newly diagnosed unilateral supratentorial GB, (c) GB without immunohistochemical staining for IDH1‐R132H, (d) tumor resected, (e) no intraoperative chemotherapy, (f) first‐line treatment with complete concomitant chemoradiotherapy according to the Stupp protocol [1]. The number of cycles of subsequent adjuvant chemotherapy with oral TMZ depended on tolerance and radiological response. Patients with a partial resection were excluded. Patients with a history of other tumors were also excluded.

All the patients meeting the inclusion and exclusion criteria outlined above were assigned to one of two subgroups on the basis of survival parameters:

1. LTS: patients with an overall survival (OS) of at least 30 months with a progression‐free survival (PFS) > 20 months where applicable.
2. STS: patients with an OS of < 12 months and a PFS > 3 months to ensure that they completed concomitant chemoradiotherapy.

We identified 26 patients meeting the criteria for LTS and 25 patients meeting the criteria for STS.

DIA‐MS was performed on 50 tumor samples (STS = 24, LTS = 26) and 30 serum samples (STS = 16, LTS = 14). One tumor sample could not be analyzed due to a high rate of necrosis and 21 patients had no preoperative serum sample. Two replicates of each sample were analyzed, with tumor and serum samples from a given individual injected in a random order. The raw data were processed using DIA‐NN with RT‐dependent cross‐run normalization and then log_2_‐transformed. For each protein, missing values were imputed as 1/5 the minimum expression value and proteins for which more than 30% of the data were missing were excluded from the analysis. Principal component analysis (PCA) was used to identify outliers. The significance analysis of microarray (SAM) method was used with the “samr” R package to identify proteins displaying significant differential expression between the STS and LTS groups. Proteins with a SAM *q*‐value < 5% were considered to display significant differential expression and therefore to be proteins of interest. Functional enrichment analyses and validations of prognostic value were then performed for these proteins.

### 2.2 Ethics approval

The study was conducted in accordance with the Declaration of Helsinki. The FGB was declared to the French Ministry of Health and Research (declaration number: DC‐2011‐1467, cession authorization number: AC‐2017‐2993, BRIF (bioresource research impact factor) number: BB‐0033‐00093). The protocols and regulations of the FGB were approved by the CPP OUEST II ethics committee (CB 2012/02, date of approval: 20 December 2011) and the CNIL (“Commission Nationale de l'Informatique et des Libertés”, the French national data protection authority, no. 1476342, date of approval: 10 October 2011). All adult GB patients included in this study signed an informed consent form for the inclusion of their clinical data and samples in the FGB biobank.

### 2.3 Collection of clinical data and biological material

Baseline characteristics, such as age, sex, preoperative KPS, tumor location, extent of resection (EOR) and Stupp protocol regimen, were collected from medical records. EOR was recorded by the surgeon performing the operation or was evaluated by a neuroradiologist from a postoperative MRI scan performed within 48 h of surgery. EOR was classified as gross total (GTR; 100%), subtotal (STR; ≥ 90%), or partial (PR; < 90%). OS was defined as the interval from the date of initial surgery to the date of the last follow‐up or death. PFS was measured from the date of initial surgery to the date of first progression. Formalin‐fixed paraffin‐embedded (FFPE) blocks of GB tissue were stored at room temperature. Serum aliquots were prepared from blood collected before surgery and then stored at −80 °C.

### 2.4 Analysis of *MGMT* promoter methylation

DNA was extracted from GB FFPE tissue sections with the QIAmp DNA FFPE tissue kit (Qiagen, Courtaboeuf, France). The methylation status of the promoter of the *MGMT* gene was assessed by pyrosequencing with the PyroMark kit (Qiagen) on five representative CpG islands previously treated by bisulfitation [34]. The average of the five CpG methylation statuses (ratio C/T incorporation) was compared to the threshold (positivity > 9%) in order to define the methylation status for each sample [34].

### 2.5 Sample preparation and protein extraction

Tumor and serum samples from STS and LTS with IDH‐wildtype GB were processed as previously described [35, 36]. Briefly, GB FFPE tissue blocks were cut into 20 μm sections, which were then mounted on glass slides and compared with hematoxylin and eosin‐stained slides from the same block to identify tumor‐rich tissue regions. These specific regions were removed from the glass slides with a scalpel and placed in microtubes. Paraffin was removed from the samples by washes in xylene and ethanol, and the samples were then solubilized in Rapigest extraction buffer (Waters, Milford, MA, USA) for reduction, cysteine alkylation and trypsinization. The quality of serum samples was checked against a hemolysis reference palette, and the samples were then depleted of the 14 most abundant proteins by antibody‐based depletion with the Human 14 Multiple Affinity Removal System, MARS‐Hu 14 (Agilent Technologies, Santa Clara, CA, USA). They were then reduced, alkylated and subjected to protein digestion to generate peptides. All samples were then stored at −80 °C until analysis by DIA‐MS.

### 2.6 DIA‐MS

Each tumor or serum sample (200 ng) was analyzed by a LC–MS/MS with a nano‐HPLC UHPLC system (Bruker Daltonik GmbH, Bremen, Germany) based on an Aurora series reverse‐phase C18 column (25 cm × 75 μm i.d., 1.6 μm C18; IonOpticks, Fitzroy, Vic., Australia) heated to 50 °C and coupled to a TimsTOF Pro2 (Bruker Daltonik GmbH). A gradient of 2–35% buffer B (0.1% formic acid in acetonitrile) over 60 min was used with 0.1% formic acid in water as mobile phase A. The total run time, including a ramp up to 35–95% buffer B to clean the column and prep for the next sample, was 90 min. Measurements were acquired in DIA‐PASEF mode (data independent acquisition parallel accumulation serial fragmentation). We analyzed 200 ng of peptides in DIA‐PASEF mode over a 60‐min gradient. The default *m/z* range was 400–1201, with an IM range of 0.6–1.43 1/K0 [V s·cm^−2^], corresponding to an estimated cycle time of 1.80 s. Default settings were also used for DIA‐PASEF windows and collision energy, with a base of 0.85 1/K0 [V s·cm^−2^] set at 20 eV and 1.30 1/K0 [V s·cm^−2^] set at 59 eV. TIMS and mass calibration were performed linearly with three calibrating ions at 622, 922, and 1222 *m/z* (Agilent Technologies) at different ranges to match the Δ1/K0 of the corresponding set of ranges. Mass spectrometry data were analyzed with DIA‐NN v1.8, by searches against the reviewed Human Uniprot database (retrieved 4/21), with the software in library‐free mode. The match‐between‐runs feature was used for all analyses and the output (precursor) was filtered at an FDR of 0.01. Retention time alignment and correction for mass accuracy were performed automatically.

### 2.7 Functional enrichment analysis

The biological functions and potential signaling pathways of the tumor and serum proteins of interest, displaying significant differential expression between the STS and LTS groups with the SAM method, were analyzed with metascape (http://metascape.org) [37].

### 2.8 Hub proteins and module analysis

Protein–protein interaction (PPI) network analysis was performed with the STRING database version 11.5 [38]. A confidence score of ≥ 0.40 was selected for the construction of the PPI network of significantly differentially expressed proteins in cytoscape version 3.9.0. We used CytoHubba [39], a cytoscape plugin, to explore PPI network hub proteins with two algorithms: maximal clique centrality (MCC) and density of maximum neighborhood component (DMNC). The top 10 proteins identified with each topological algorithm were considered to be hub proteins. Molecular Complex Detection (MCODE; version 1.5.1) was performed in cytoscape [40] to identify the most significant modules in the PPI network, considering MCODE scores > 3, degree cutoff = 2, node score cutoff = 0.2, max depth = 100 and *k*‐score = 2. Functional enrichment analyses were performed with metascape for the proteins in the modules.

### 2.9 Immunohistochemistry

Immunohistochemistry (IHC) was performed on 4 μm sections from GB FFPE tissue blocks with an automated Leica BOND III (Leica Biosystems, Nanterre, France) in accordance with the manufacturer's instructions. Antibodies diluted in BOND Primary Antibody Diluent (Leica) were used as follows: FABP7 and TJAP1 (Bio‐Techne, Rennes, France) were used at a concentration of 1.5 μg·mL^−1^ for EDTA antigen retrieval; AHSP (Bio‐Techne) was used at a concentration of 1 μg·mL^−1^ for citrate antigen retrieval. Primary antibody binding to GB tissue sections was visualized with BOND Polymer Refine Detection (Leica). Digital images were captured with an Aperio CS2 scanner (Leica) with a 20× objective and were analyzed with aperio imagescope software v12.3.2.8009 (Leica).

### 2.10 Validation of the prognostic value of proteins of interest

The TCGA‐GB database (https://portal.gdc.cancer.gov/) was used to validate the prognostic value of the tumor proteins of interest. The following inclusion criteria were selected from the clinical data of this dataset: (a) patient aged ≥ 18 years, (b) newly diagnosed GB, (c) GB with IDH‐wildtype status, and (d) treatment with radiotherapy and chemotherapy (TMZ or alkylating agent). Patients with early mortality (30 days) for whom death could be attributed to postoperative complications were excluded. Based on these criteria, we selected 99 patients from the TCGA dataset. DIA‐MS data for serum samples from 96 IDH‐wildtype GB patients undergoing surgery at Angers University Hospital and included in the FGB were used to validate the prognostic value of the serum proteins of interest. The same inclusion and exclusion criteria described above were used for the selection of these 96 IDH‐wildtype GB patients.

### 2.11 Statistical analysis

Statistical analyses were performed with r software (version 4.1.0; https://www.R‐project.org/), with the adjustment of *P*‐values by the Benjamini–Hochberg (BH) method for multiple testing and values of *P* < 0.05 considered significant. Differences between the STS and LTS groups were evaluated in overall Chi‐squared or Fisher's exact tests. Univariate Cox regression analysis was performed to assess the prognostic value of the tumor and serum proteins of interest, which were analyzed as both continuous and dichotomous variables. Dichotomization was achieved by a median split or the use of an optimal cutoff set according to the maximally selected rank statistics from the “maxstat” R package. Survival curves were plotted according to the Kaplan–Meier method and were compared in log‐rank tests. The minimizing approximated information criteria (MIC) method from the “coxphMIC” R package [41] was used to select the serum proteins most useful for the construction of a prognostic blood score. The multivariate Cox regression model was used to evaluate the blood score as an independent predictor of survival. Variables with raw *P*‐values < 0.05 in univariate analysis were included in the multivariate model unless they were correlated with each other. The demographic variables age and sex, which differed significantly between the STS and LTS groups, were forced into the models, regardless of their significance. The performance of the prognostic blood score was assessed by plotting the receiver operating characteristic (ROC) curve and calculating the area under the curve (AUC) with the “survivalROC” R package. An AUC value ≥ 0.75 was considered significant, and values ≥ 0.60 were considered acceptable for prediction.

## 3 Results

### 3.1 Patient characteristics

The baseline characteristics of the 51 selected IDH‐wildtype GB patients are shown in Table 1. These patients were grouped according to survival: STS (*n* = 25) and LTS (*n* = 26) (Table 1). PFS and OS differed significantly between these two groups. Median PFS was 5.8 months [95% CI: 5.0–6.8] in the STS group and 37.3 months [95% CI: 30.9–54.3] in the LTS group (*P* < 0.001). Median OS was 9.4 months [95% CI: 8.1–10.8] in the STS group and 47.1 months [95% CI: 40.9–89.7] in the LTS group (*P* < 0.001). The two groups also differed significantly in terms of age (*P* = 0.036), sex (*P* = 0.003), *MGMT* methylation status (*P* < 0.001), and TMZ consolidation treatment (*P* < 0.001). The patients in the STS group were older and more likely to be male patients with *MGMT*‐unmethylated GB and a short TMZ consolidation treatment. Other variables, such as preoperative KPS (*P* = 1.000), tumor laterality (*P* = 0.691), extent of the tumor (*P* = 0.180) and EOR (*P* = 0.492) did not differ significantly between the two groups.

**Table 1:** Demographic and clinical characteristics of STS and LTS with IDH‐wildtype GB treated with a first‐line Stupp's regimen. *Significant difference. EOR, extent of resection; GB, glioblastoma; GTR, gross total resection (100%); KPS, Karnofsky performance score; LTS, long‐term survivors; *MGMT*, O(6)‐methylguanine methyltransferase; NA, not applicable; OS, overall survival; PFS, progression‐free survival; STR, subtotal resection (≥ 90%); STS, short‐term survivors; TMZ, temozolomide.

	STS	LTS	*P*‐value
Number	25 (49%)	26 (51%)	
Age (years)			
Median (range)	64 (40–76)	60 (36–81)	0.036*
≤ 60	9 (36%)	17 (65%)	
> 60	16 (64%)	9 (35%)	
Sex			
Male	22 (88%)	13 (50%)	0.003*
Female	3 (12%)	13 (50%)	
Preoperative KPS			
< 70	1 (4%)	1 (4%)	1.000
≥ 70	23 (92%)	21 (81%)	
Unknown	1 (4%)	4 (15%)	
Tumor laterality			
Right	15 (60%)	17 (65%)	0.691
Left	10 (40%)	9 (35%)	
Extent of tumor			
Unilobar	16 (64%)	21 (81%)	0.180
Multilobar	9 (36%)	5 (19%)	
EOR			
STR	12 (48%)	10 (38%)	0.492
GTR	13 (52%)	16 (62%)	
*MGMT* methylation status			
Without methylation	18 (72%)	6 (23%)	< 0.001*
With methylation	6 (24%)	20 (77%)	
NA	1 (4%)	0 (0%)	
TMZ consolidation			
< 6 cycles	25 (100%)	10 (38%)	< 0.001*
≥ 6 cycles	0 (0%)	16 (62%)	
Survival outcome			
PFS			
Median (months) [95% CI]	5.8 [5.0–6.8]	37.3 [30.9–54.3]	< 0.001*
OS			
Median (months) [95% CI]	9.4 [8.1–10.8]	47.1 [40.9–89.7]	< 0.001*

### 3.2 Identification of proteins of interest differentially expressed between the STS and LTS groups

Fifty tumor samples (STS = 24, LTS = 26) and 30 serum samples (STS = 16, LTS = 14) were analyzed by the DIA‐MS. In total, 6934 proteins (5422 normalized proteins) and 953 proteins (826 normalized proteins) were identified in tumor and serum samples, respectively (Tables S1 and S2). PCA identified no outliers among the tumor samples and one outlier among the serum samples, which was excluded from the analysis. Three tumor proteins and 26 serum proteins were found to display significant differential expression between the STS and LTS groups (Table 2, Tables S1 and S2). These differentially expressed proteins were considered to be proteins of interest. The three tumor proteins of interest (AHSP, FABP7 and TJAP1) were downregulated in the STS group (Table 2) and were not identified in the proteome of serum samples from GB patients. An example IHC analysis for these proteins on GB tissues from the STS and LTS groups is presented in Fig. S1. The 26 serum proteins of interest were upregulated in the STS group (Table 2); 23 of these proteins were also identified in the proteome of the tumor samples, but were similarly expressed in the STS and LTS groups.

**Table 2:** List of proteins of interest from tumor and serum samples displaying differential expression between the STS and LTS groups. GB, glioblastoma; LTS, long‐term survivors; STS, short‐term survivors.

Gene symbol	Protein	Fold change (STS/LTS)	*Q*‐value (%)
GB tissue			
*FABP7*	Fatty acid‐binding protein 7	0.37	0.00
*TJAP1*	Tight junction associated protein 1	0.29	0.00
*AHSP*	Alpha hemoglobin stabilizing protein	0.19	0.00
Serum			
*EEF1G*	Eukaryotic translation elongation factor 1 gamma	6.02	0.00
*CCN2*	Cellular communication network factor 2	4.75	0.00
*EPB41*	Erythrocyte membrane protein band 4.1	4.48	0.00
*USP14*	Ubiquitin‐specific peptidase 14	3.76	0.00
*TXNL1*	Thioredoxin‐like 1	3.63	0.00
*TBCA*	Tubulin‐folding cofactor A	2.47	0.00
*HBD*	Hemoglobin subunit delta	2.14	0.00
*TXNDC17*	Thioredoxin domain‐containing 17	2.04	0.00
*RNH1*	Ribonuclease/angiogenin inhibitor 1	1.95	0.00
*SOD1*	Superoxide dismutase 1	1.91	0.00
*PRDX1*	Peroxiredoxin 1	1.87	0.00
*STX7*	Syntaxin 7	4.92	3.99
*IL1RL1*	Interleukin 1 receptor‐like 1	3.97	3.99
*HPRT1*	Hypoxanthine phosphoribosyltransferase 1	3.02	3.99
*CAPG*	Capping actin protein, gelsolin like	2.67	3.99
*AK1*	Adenylate kinase 1	2.19	3.99
*MMP3*	Matrix metallopeptidase 3	2.06	3.99
*CA2*	Carbonic anhydrase 2	2.02	3.99
*ZYX*	Zyxin	2.00	3.99
*BPGM*	Bisphosphoglycerate mutase	1.85	3.99
*CAT*	Catalase	1.76	3.99
*PRDX2*	Peroxiredoxin 2	1.76	3.99
*FKBP1A*	FKBP prolyl isomerase 1A	1.68	3.99
*SH3BGRL*	SH3 domain binding glutamate‐rich protein like	1.64	3.99
*YWHAE*	Tyrosine 3‐monooxygenase/tryptophan 5‐monooxygenase activation protein epsilon	1.57	3.99
*MDH1*	Malate dehydrogenase 1	1.52	3.99

### 3.3 Functional enrichment analysis on proteins of interest

**Fig. 1:**
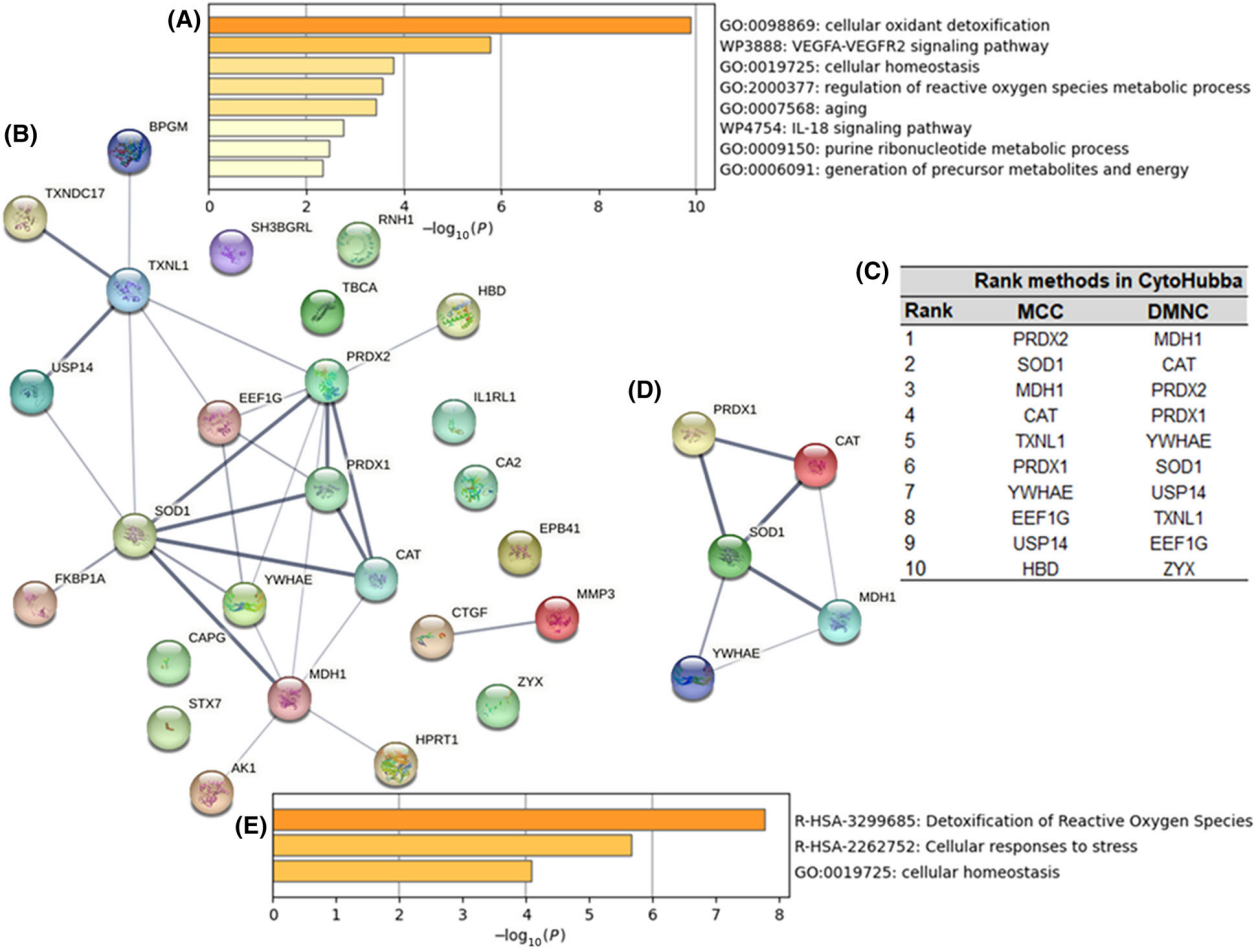
Functional enrichment analysis and identification of hub proteins and key modules. (A) metascape bar graph of functional enrichment terms of the 26 serum proteins upregulated in the STS group (colored by *P*‐values). (B) PPI network analysis with the STRING database on the 26 serum proteins upregulated in the STS group. Line thickness indicates the strength of data support. (C) Identification of 11 hub serum proteins with two CytoHubba algorithms: MCC and DMNC. (D, E) Identification of the most significant module in the PPI network with MCODE in cytoscape (score of the module = 3.5) (D) and the metascape functional enrichment analysis of this module (E). DMNC, density of maximum neighborhood component; MCC, maximal clique centrality; PPI, protein–protein interaction; STS, short‐term survivors.

metascape analysis of the three tumor proteins of interest indicated that FABP7, TJAP1 and AHSP were associated with fatty‐acid transport (GO:0015908), Golgi organization (GO:0007030) and hemoglobin metabolic process (GO:0020027), respectively. The 26 serum proteins of interest were associated with six gene ontology (GO) biological processes: cellular oxidant detoxification (GO:0098869), cellular homeostasis (GO:0019725), regulation of reactive oxygen species metabolic process (GO:2000377), aging (GO:0007568), purine ribonucleotide metabolic process (GO:0009150) and generation of precursor metabolites and energy (GO:0006091) (Fig. 1A). They were also associated with two WikiPathways: VEGFA‐VEGFR2 signaling pathway (WP3888) and IL‐18 signaling pathway (WP4754) (Fig. 1A).

### 3.4 Identification of hub proteins and key modules

The three tumor proteins of interest were subjected to PPI network analysis. No association was found between these three proteins. The similar analysis on the 26 serum proteins of interest identified 26 nodes and 27 edges (Fig. 1B). Topological feature analysis on these serum proteins of interest led to the identification of 11 hub proteins: SOD1, PRDX2, TXNL1, MDH1, PRDX1, EEF1G, YWHAE, CAT, USP14, ZYX and HBD (Fig. 1C). One significant module containing five hub proteins (SOD1, MDH1, PRDX1, YWHAE and CAT) was extracted with the MCODE plugin tools of cytoscape applied to the PPI network (Fig. 1D). Functional analysis indicated that this module was associated with a GO biological process, cellular homeostasis (GO: 0019725), and two reactome gene sets: detoxification of reactive oxygen species (R‐HSA‐3299685) and cellular responses to stress (R‐HSA‐2262752) (Fig. 1E).

### 3.5 Validation of the prognostic value of the tumor proteins of interest

**Fig. 2:**
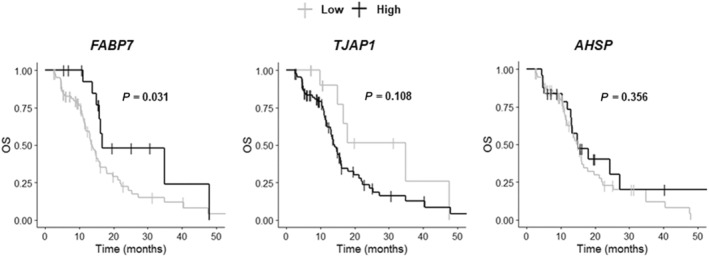
Prognostic value of *FABP7*, *TJAP1* and *AHSP* gene expression based on an analysis of the TCGA‐GB transcriptomic dataset (https://portal.gdc.cancer.gov/). The log‐rank test was performed to determine statistical significance (*P* < 0.05). OS, overall survival.

The TCGA‐GB transcriptomic dataset was used to validate the prognostic value of the three tumor proteins of interest (AHSP, FABP7 and TJAP1) at the mRNA level. Univariate Cox analysis for OS of mRNA levels for *FABP7*, *TJAP1* and *AHSP*, dichotomized with the optimal cutoff, indicated that low levels of *FABP7* expression were correlated with significantly shorter OS in IDH‐wildtype GB patients (*P* = 0.037) (Table S3). The levels of *TJAP1* and *AHSP* expression were not relevant to survival (*P* = 0.119 and *P* = 0.351, respectively) (Table S3). Figure 2 shows the corresponding Kaplan–Meier curves. The evaluation of *FABP7*, *TJAP1* and *AHSP* expression levels as continuous or dichotomous variables based on the median cutoff yielded no significant results in univariate Cox model analysis (data not shown).

### 3.6 Validation of the prognostic value of the serum proteins of interest

**Fig. 3:**
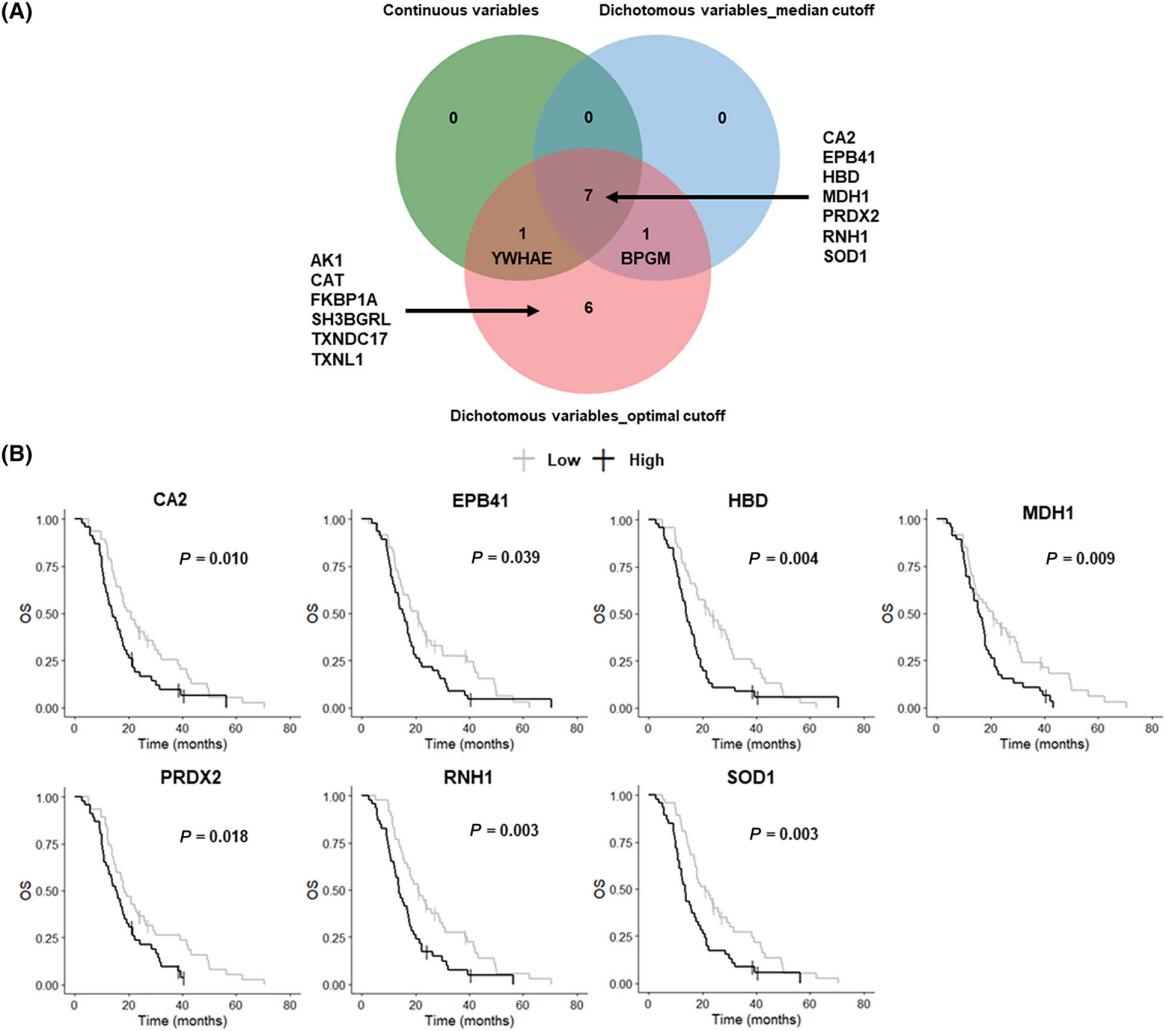
Prognostic value of the serum proteins of interest based on a serum proteome dataset for 96 IDH‐wildtype GB patients treated with a first‐line Stupp's regimen. PCA on the 96 serum samples identified three outliers, which were excluded from the analysis. (A) Venn diagram identifying the seven serum proteins associated with OS common to all three methods of univariate Cox regression analysis used, based on continuous variables, dichotomous variables with the median cutoff, and dichotomous variables with the optimal cutoff. (B) Kaplan–Meier curves for the seven serum proteins identified in the analysis of dichotomous variables based on the median cutoff. The log‐rank test was performed to determine statistical significance (*P* < 0.05). OS, overall survival; PCA, principal component analysis.

The serum proteome data for a cohort of 96 IDH‐wildtype GB patients treated with a first‐line Stupp's regimen was used to assess the prognostic value of the 26 serum proteins upregulated in the STS group. Six proteins (EEF1G, CAPG, CTGF, HPRT1, IL1RL1 and USP14) were not analyzed because more than 30% of the data were missing, and one protein, TBCA, was not identified. Furthermore, PCA on the 96 serum samples from GB patients identified three outliers, which were excluded from the analysis. The results of univariate Cox regression analysis for OS on the 19 serum proteins differed according to the treatment of these proteins as continuous variables or dichotomous variables defined according to the median or the optimal cutoff (Fig. 3A and Table S4). High levels were significantly associated with a poor OS for 8 of 19 proteins considered as continuous variables, 8 of 19 considered as dichotomous variables with the median cutoff, and 15 of 19 proteins considered as dichotomous variables with the optimal cutoff. High levels of seven of these proteins were consistently associated with a poor OS in all three methods of univariate Cox regression analysis: CA2, EPB41, HBD, MDH1, PRDX2, RNH1 and SOD1 (Fig. 3B). These seven proteins were used to define the best prognostic blood score.

### 3.7 Definition of a prognostic blood score and assessment of its performance

The MIC method was used to select the most useful of the seven proteins associated with OS for predictive purposes. Two proteins were identified: MDH1 and RNH1. The LASSO regression method was also used and identified five proteins: CA2, HBD, MDH1, RNH1 and SOD1. Based on these results, the following prognostic blood score scale was defined: score = 0, patients with low levels of MDH1 and RNH1 (*n* = 32); score = 1, patients with high levels of either MDH1 or RNH1 (*n* = 30) and score = 2, patients with high levels of both MDH1 and RNH1 (*n* = 31).

**Fig. 4:**
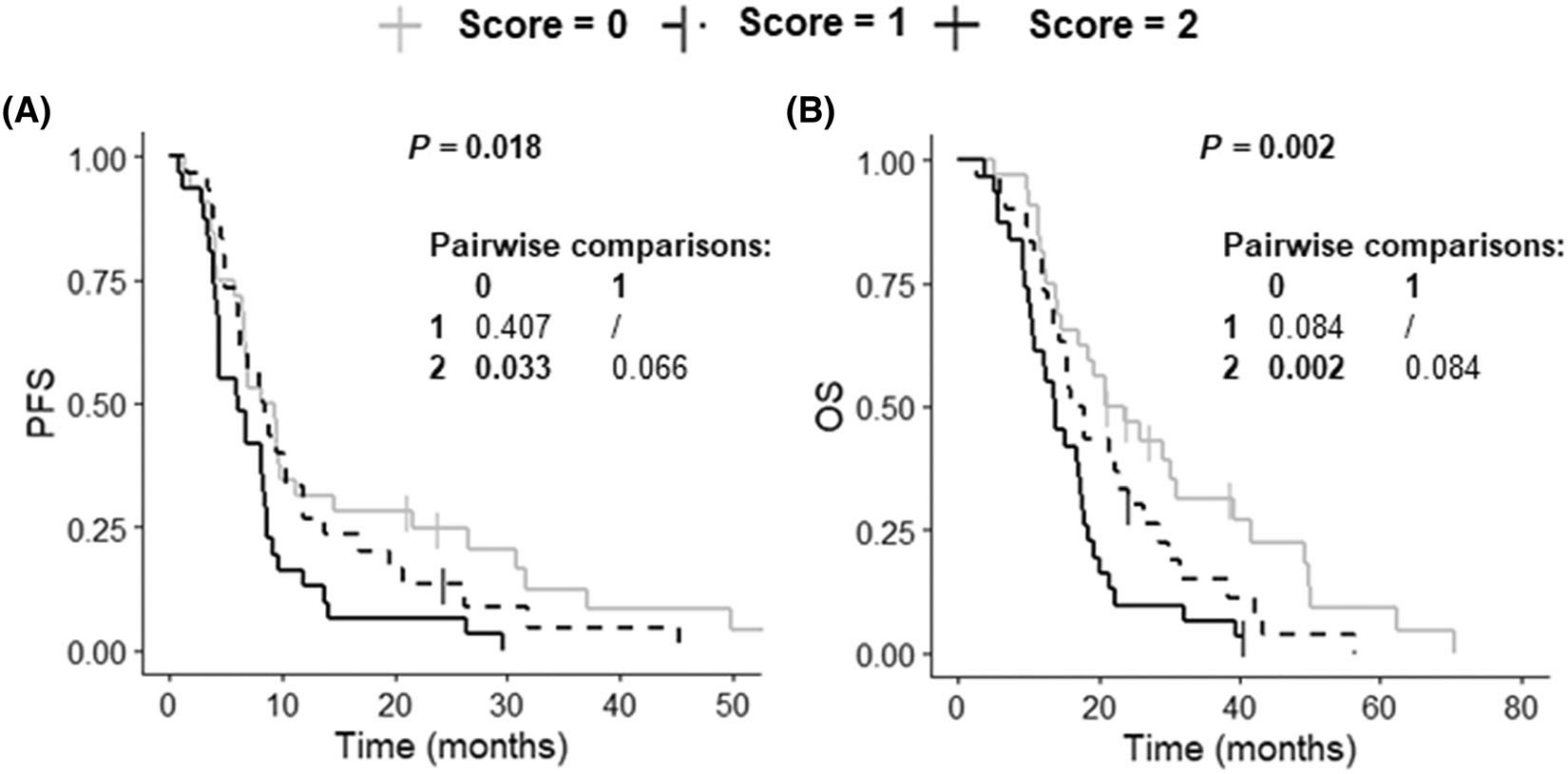
Kaplan–Meier curves for survival stratified by prognostic blood score (A: PFS; B: OS). Score = 0, patients with low levels of MDH1 and RNH1, score = 1, patients with high levels of either MDH1 or RNH1 and score = 2, patients with high levels of both MDH1 and RNH1. The log‐rank test was performed to determine statistical significance (*P* < 0.05). OS, overall survival; PFS, progression‐free survival.

The 93 IDH‐wildtype GB patients had a median PFS of 8.0 months (95% CI: 6.4–8.8) and a median OS of 17.1 months (95% CI: 14.4–20.2). The prognostic blood score was significantly associated with PFS and OS in univariate Cox regression analysis (Table S5). This score remained an independent prognostic factor for PFS and OS after adjustment for other variables, including age, sex, KPS and TMZ, in multivariate Cox regression analysis (Table 3). The PFS of patients with high serum levels of both MDH1 and RNH1 (6.0 months (95% CI: 4.3–8.6)) did not differ significantly from that of patients with high serum levels of either MDH1 or RNH1 (8.4 months (95% CI: 6.2–11.9); *post hoc P* = 0.066) but was significantly shorter than that of patients with low serum levels of MDH1 and RNH1 (8.7 months (95% CI: 6.6–14.5); *post hoc P* = 0.033) (Fig. 4A). The OS of patients with high serum levels of both MDH1 and RNH1 (13.9 months (95% CI: 10.6–17.8)) was also not significantly different to that of patients with high serum levels of either MDH1 or RNH1 (16.9 months (95% CI: 14.4–26.4); *post hoc P* = 0.084) but significantly shorter than that of patients with low levels of MDH1 and RNH1 (22.3 months (95% CI: 17.1–39.1); *post hoc P* = 0.002) (Fig. 4B).

**Table 3:** Multivariate Cox regression analysis of factors associated with PFS and OS in 93 IDH‐wildtype GB patients treated with a first‐line Stupp's regimen. *Significant difference. CI, confidence interval; GB, glioblastoma; HR, hazard ratio; KPS, Karnofsky performance score; OS, overall survival; PFS, progression‐free survival; TMZ, temozolomide.

Variable	PFS			OS		
	HR	95% CI	*P*‐value	HR	95% CI	*P*‐value
Age (≥ 63 years)	0.63	0.40–1.01	0.055	1.03	0.65–1.62	0.905
Sex (female)	0.82	0.51–1.32	0.413	0.75	0.45–1.25	0.273
KPS (> 80%)	0.70	0.40–1.22	0.204	0.34	0.19–0.60	< 0.001*
TMZ consolidation (≥ 6 cycles)	0.07	0.04–0.15	< 0.001*	0.25	0.15–0.44	< 0.001*
Prognostic blood score						
Score = 0	1			1		
Score = 1	1.34	0.77–2.34	0.306	2.00	1.11–3.58	0.020*
Score = 2	1.93	1.11–3.36	0.019*	2.49	1.41–4.40	0.002*

With OS as the endpoint, the AUC for prognostic blood score was 0.61 at 1 year, 0.68 at 3 years, and 0.80 at 4 years, indicating that combined MDH1 and RNH1 levels were of value for predicting the OS of GB patients. This combination also had predictive value for the PFS of GB patients, with an AUC of 0.61 at 1 year, 0.76 at 3 years, and 0.84 at 4 years.

## 4 Discussion

Short‐term survivors and LTS with IDH‐wildtype GB have very different survival profiles but, surprisingly, only a few tumor and serum proteins were found to be differentially expressed between these two groups in DIA‐MS‐based proteomics analyses. For tumor samples, only three of the 5422 normalized proteins displayed significant downregulation in the STS group: FABP7, TJAP1 and AHSP. No functional enrichment was observed for these three proteins. FABP7, a member of the fatty acid‐binding proteins (FABPs) family also known as brain‐typed FABP (B‐FABP) or brain lipid‐binding protein (BLBP), facilitates the transportation of fatty acids across the membranes of various cell organelles, regulating their metabolism and other physiological activities. FABP7 is abundant in several types of glial cells, such as astrocytes and oligodendrocyte progenitor cells, during brain development [42]. TJAP1, also known as tight junction protein 4 (TJP4) or protein incorporated later into tight junctions (TJ) (Pilt), is a peripheral membrane protein located in TJ complexes; it was first described in 2001, but its function remains unclear [43]. Yan et al. [44] found that miR‐132/212 specifically inhibited the expression of TJAP1 and Claudin‐1 by directly binding to their 3′‐UTR mRNA regions, leading to an increase in blood–brain barrier integrity. AHSP is a small chaperone protein that binds specifically to the free alpha‐globin chain of hemoglobin (αHb). Its main function is thought to be regulation of the stability, folding, and assembly of the αHb subunit. AHSP plays an important role in the physiological process of erythropoiesis and may also modulate pathological conditions, such α‐thalassemia‐like syndromes [45]. Prognostic value was validated at gene level with the TCGA‐GB dataset for FABP7, but not for TJAP1 and AHSP. It was not possible to use published proteomics datasets such as those reported by Wang et al. [46] and Yanovich‐Arad et al. [19] because the tandem mass tag approach they used does not allow for the exclusion of proteome data for healthy tissues or IDH‐mutated GB samples from calculations. A label‐free approach, such as that used here, would have made it possible to recover data from the samples of interest. For serum samples, 26 of the 826 normalized proteins were significantly upregulated in the STS group and 11 of these proteins were hub proteins. These proteins were principally associated with cellular oxidant detoxification and metabolic processes. The prognostic value of most of these proteins was validated with a serum proteome dataset for 93 IDH‐wildtype GB patients given the standard first‐line treatment. Two circulating proteins were of particular interest: MDH1 and RNH1. A prognostic blood score based on the levels of these two proteins was developed. GB patients with high blood score (i.e., with high levels of MDH1 and RNH1) had worse OS and PFS than those with low score. Multivariate analysis indicated that high blood score was an independent predictor of shorter PFS and OS. The prognostic performance of the blood score for long‐term mortality at 4 years was good, with an AUC of 0.80.

**Fig. 5:**
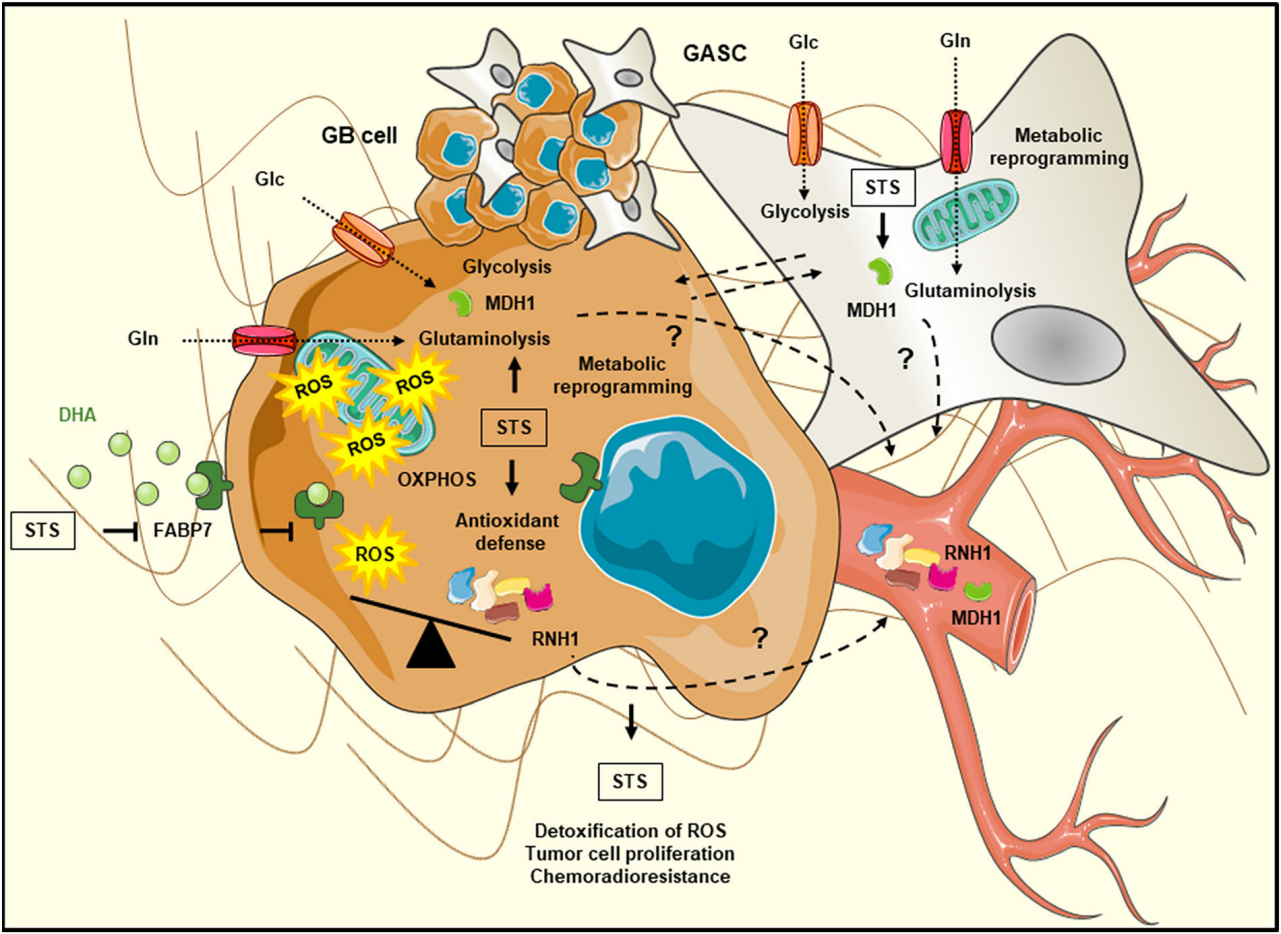
Schematic representation of the putative mechanisms maintaining low ROS levels in STS with IDH‐wildtype GB, which would promote tumor cell proliferation and chemoradioresistance: low levels of tumor FABP7 expression to decrease DHA uptake and the production of large amounts of serum proteins associated with cellular oxidant detoxification and metabolic processes, such as RNH1 and MDH1. “?”: We can hypothesize that the upregulation of MDH1 in the serum of STS results from a need for high levels of production in GB cells and/or GASCs to induce metabolic reprogramming by increasing glutaminolysis and/or glycolysis with respect to OXPHOS, so as to decrease ROS production, and, thus oxidative stress. We can also hypothesize that the upregulation of serum RNH1 results from high levels of production in GB cells to prevent ROS accumulation. DHA, docosahexaenoic acid; GASC, glioma‐associated stromal cell; GB, glioblastoma; Glc, glucose; Gln, glutamine; OXPHOS, oxidative phosphorylation; ROS, reactive oxygen species; STS, short‐term survivors.

Published data on the three potential prognostic biomarkers identified, FABP7, MDH1 and RNH1, indicate that high levels of FABP7 are associated with a poor prognosis for patients with various types of tumors, including malignant gliomas [42, 47]. This is contrary to our findings. However, FABP7 may have several different roles in cells, depending on the ligand it binds. Polyunsaturated fatty acids, such as arachidonic acid (AA) and docosahexaenoic acid (DHA), are the preferred ligands of FABP7, with DHA having a binding affinity for FABP7 four times higher than that of AA [48]. It has been suggested that an AA‐rich tumor microenvironment promotes FABP7‐expressing GB cell growth whereas a DHA‐rich microenvironment may inhibit tumor infiltration due to higher levels of DHA uptake and use [48, 49]. Xie et al. [50] recently identified FABP7 as a new potential biomarker for predicting the response to neoadjuvant chemotherapy for breast cancer. They found that high levels of FABP7 were associated with a better response to chemotherapy in patients with estrogen receptor‐negative breast cancer, resulting in longer recurrence‐free survival. The molecular mechanisms by which FABP7 contributes to chemosensitivity require clarification. They may involve an increase of DHA uptake in cancer cells, as already reported for GB neural stem‐like cells [48]. In accordance with this hypothesis, several studies have indicated that DHA not only stimulates apoptosis *per se* but also increases the responsiveness of cancer cells, including GB cells, to chemotherapy drugs by generating reactive oxygen species (ROS) [51, 52, 53, 54]. Furthermore, multiple clinical trials have demonstrated that DHA (fish oil) supplementation during chemotherapy reduces the adverse effects of the drugs used and has beneficial effects on immune function, bone health, inflammation, tumor‐induced cachexia and the efficacy of chemotherapy [55, 56, 57]. Given these data, we can hypothesize that the lower levels of FABP7 in the GB tissues of STS underlie chemoresistance through a mechanism involving decreases in DHA uptake and the prevention of high ROS levels (Fig. 5). MDH1, one of the 11 hub proteins identified, is the cytosolic form of MDH, an important enzyme in cancer metabolism. *MDH1* is amplified in a broad spectrum of cancers and this amplification is correlated with poor prognosis [58, 59]. Hanse et al. [58] showed that MDH1 plays a crucial role in replenishing cytosolic NAD to support increases in glycolysis during tumor proliferation. MDH1 is also involved in the glutamine catabolic pathway. Wang et al. [60] reported that *MDH1* knockdown in pancreatic ductal adenocarcinoma cells (PDACs) represses mitochondrial respiration and inhibits glutamine metabolism, sensitizing PDACs to oxidative stress and preventing cell proliferation. We can hypothesize that the upregulation of MDH1 in the serum of STS results from a need for high levels of production in GB cells to induce metabolic reprogramming by increasing glutaminolysis and/or glycolysis (i.e., the Warburg effect) with respect to oxidative phosphorylation (OXPHOS), so as to decrease ROS production and, thus, oxidative stress (Fig. 5). The metabolic reprogramming of GB cells to adapt to high levels of ROS has already been described [61]. The large amounts of MDH1 in the serum of STS may also result from production by the stromal cells in the tumor microenvironment. Recent studies have shown that cancer‐associated fibroblasts (CAFs) may display an activation of metabolism, with enhanced glycolytic activity and a major role in the regulation of cancer cell metabolism [62]. In previous studies, we isolated glioma‐associated stromal cells (GASCs) from the peritumoral microenvironment of GB that had phenotypic and functional properties similar to those of mesenchymal stem cells and CAFs [63, 64]. These GASCs, which are of prognostic value in glioma, may undergo metabolic reprogramming and induce the metabolic reprogramming of GB cells through MDH1 (Fig. 5). RNH1 is an inhibitor of secretory and intracellular ribonucleases. Conflicting data have been obtained concerning the pro‐ or anti‐oncogenic nature of RNH1, but several studies have reported it to have antioxidant and redox homeostatic effects in various malignant and non‐malignant cell types [65, 66, 67]. Furthermore, Kun et al. [68] showed that high levels of expression for five driver genes, including *RNH1*, were associated with poor prognosis in patients with GB. The large amounts of RNH1 in the serum of STS may result from high levels of production in GB cells to decrease ROS production, as hypothesized for MDH1.

## 5 Limitations

This study has several limitations. The small fraction of LTS with IDH‐wildtype GB made it difficult to obtain a large cohort of biological samples. Furthermore, there were too few mirror tumor samples available to combine proteomics with other ‐omics technologies, such as genomics, transcriptomics and metabolomics. In addition, the technique used for assessment of the IDH status of tumors was immunohistochemistry for IDH1‐R132H. Sanger sequencing of the IDH1/2 genes was not systematically performed. It was also not possible to assess the impact of *MGMT* methylation status on the performance of the blood score because of the large amounts of missing data in the cohort used. An analysis of *MGMT* methylation status is not mandatory for routine pathology reports in the FGB network because of its minimal relevance to clinical decision‐making for first‐line treatment. Another limitation is the lack of formal volumetric analysis to measure preoperative tumor volume and residual volume. The lack of consensus for a clearly defined optimal statistical method for cutoff selection for protein analyses is also a limitation. We used biomarker‐oriented (median cutoff) and outcome‐oriented (optimal cutoff) approaches. Outcome‐oriented methods are generally expected to yield better statistical indicators than biomarker‐oriented methods [69]. The lack of information about the levels of the proteins of interest in brain tissue or serum from healthy individuals may also be considered a weakness of this study. We used the UALCAN portal (http://ualcan.path.uab.edu) [70, 71] to compare the levels of the three tumor proteins of interest in GB and control brain tissue samples. Levels of the FABP7, TJAP1 and AHSP proteins were significantly upregulated in GB tissue samples relative to control brain tissue samples (*P* < 0.001). However, this result should be interpreted with caution, as the data were obtained from 99 treatment‐naive GB prospectively collected by the Clinical Proteomic Tumor Analysis Consortium (CPTAC), 91 of which were IDH‐wildtype GB, but the other eight were IDH‐mutated GB. The expression profiles of the 26 serum proteins displaying significant differential expression between the STS and LTS groups were explored in GB and normal serum samples with proteomics data from Arora et al. [22]. However, these proteins were not identified in this proteomics dataset, probably due to the technique used. The 4‐plex iTRAQ methodology used identified only 102 proteins in GB serum samples, whereas the DIA‐MS method we used identified 953 proteins in GB serum samples. Serum samples from healthy individuals are currently being stored at Angers University Hospital for future use in a DIA‐MS analysis comparing serum samples from patients with IDH‐wildtype GB and healthy controls.

## 6 Conclusions

This proteomics study highlights three potential biomarkers of GB prognosis: a GB tissue biomarker, FABP7, and two circulating biomarkers, MDH1 and RNH1. These biomarkers and the other serum proteins dysregulated between STS and LTS with IDH‐wildtype GB were associated principally with ROS detoxification. Cancer cells, including GB cells, have high ROS levels due to their high rates of metabolism, gene mutation and microenvironment‐associated alterations [72, 73, 74, 75, 76]. The balance between ROS production and elimination is crucial to the survival of these cells. Indeed, at low levels, ROS act as signal transducers, activating cell proliferation, migration, invasion, angiogenesis, and resistance to chemotherapy, whereas their accumulation within cells can lead to oxidative damage to proteins, lipids, RNA and DNA, eventually inducing cell death. STS with IDH‐wildtype GB may have developed different mechanisms for decreasing high levels of ROS including low levels of tumor FABP7 expression to decrease DHA uptake, the production of large amounts of proteins with antioxidant properties, such as RNH1, and the metabolic reprogramming of tumor cells or GASCs supported by high levels of MDH1 production (Fig. 5). Other proteomics studies on GB have highlighted an association between metabolic reprogramming and survival, with high OXPHOS levels linked to longer survival [19, 77]. The mechanisms by which STS maintain low levels of ROS may account for the poor efficacy of radiotherapy plus concomitant and adjuvant TMZ chemotherapy in these patients. This standard treatment depends on the induction of ROS production and several studies have indicated that the acquisition of TMZ resistance results at least partly from low levels of intracellular ROS accumulation associated with a stronger expression of ROS scavenging systems [73, 78, 79]. Various studies have tested combinations of TMZ and agents increasing ROS production and/or inhibiting antioxidant processes [72, 78]. However, despite the excellent results obtained in GB cell lines and *in vivo* models, only a few of these combinations have been tested in clinical trials, and no major progress has been made. This may be due, in part, to a lack of targeting of the redox signaling pathways potentially used by GB cells to decrease high levels of ROS. The results presented here identify the pathways driven by FABP7, MDH1 and RNH1 as promising targets for blocking ROS detoxification in potential STS with IDH‐wildtype GB.

## 7 Perspectives

The new insights highlighted by this proteomics study now require urgent validation to drive the use of novel prognostic biomarkers and the development of novel therapies for IDH‐wildtype GB. In particular, additional studies are required to determine whether the analysis of FABP7 expression in GB tissues and that of MDH1 and RNH1 in serum samples can be integrated into clinical practice for evaluations of the prognosis of patients with IDH‐wildtype GB. For example, the levels of FABP7, MDH1 and RNH1 in brain tissue or serum of healthy individuals now need to be compared with the findings for GB samples. In addition, it would be interesting to determine whether the blood score based on MDH1 and RNH1 levels is as effective as *MGMT* promoter methylation for predicting responses to standard first‐line treatment. Further studies are also required to determine whether the pathways driven by FABP7, MDH1 and RNH1 constitute promising therapeutic targets for blocking ROS detoxification to overcome resistance to chemoradiotherapy in potential GB STS. Confirmation is therefore required that the association between MDH1, RNH1 and FABP7 levels and prognosis is due to the elimination or increase in levels of ROS. The measurement of ROS levels *in vivo* and *ex vivo* is complex. Murphy et al. [80] suggested that ROS levels should not be measured in tissue homogenates or cryosections. Experiments on GB cultures derived from STS and LTS would therefore be more appropriate but are not yet available. Fresh GB tissues are currently frozen in DMSO in FGB for the development of cell‐ and animal‐based GB models. These models, and their association with clinical data, will make it possible to conduct experiments on STS and LTS cohorts, but it will take time to establish statistically appropriate cohorts, particularly for the LTS cohort. The knockdown of MDH1, RNH1, and FABP7 expression in cell‐based GB models will make it possible to investigate the specific roles of these proteins in eliminating or increasing the levels of ROS, together with their contribution to the GB phenotype in migration, invasion and colony formation assays. It would also be interesting to confirm that the association between high levels of FABP7 expression in GB tissues and a better prognosis is due to higher levels of DHA in the tumor microenvironment. DHA levels are generally ~ 50% lower in malignant glioma samples than in normal brain tissue [81]. DHA supplementation could be offered as a means of increasing these levels. Nasrollahzadeh et al. [82] have already shown that dietary supplementation with a DHA‐containing oil can effectively increase the levels of long‐chain n‐3 fatty acids in brain tumors, at least partly through an upregulation of FABP7.

## Conflict of interest

The authors declare no conflict of interest.

## Author contributions

AC contributed to conceptualization, data analysis, statistical analysis, writing—original draft, writing—review and editing; CG contributed to conceptualization, quantitative DIA‐MS data processing, writing—review and editing; HL contributed to data analysis, statistical analysis, writing—review and editing; AR contributed to histological analysis, writing—review and editing; OB contributed to storage and availability of tumor and serum samples; CH and AB contributed to sample preparation and DIA‐MS analysis; MC contributed to the *MGMT* promoter methylation analysis of LTS and STS GB tissues; PJ contributed to statistical analysis, writing—review and editing; FG contributed to quantitative DIA‐MS data processing; PM was the coordinator of the FGB; J‐ML contributed to conceptualization, statistical analysis, writing—review and editing. All the authors have read and agreed to submission of the published version of the manuscript.

### Peer review

The peer review history for this article is available at https://www.webofscience.com/api/gateway/wos/peer‐review/10.1002/1878‐0261.13668.

## Supporting information


**Fig. S1.** IHC staining for AHSP, FABP7 and TJAP1 on tumor tissues from STS and LTS with IDH‐wildtype GB.
**Table S1.** Results of the SAM method to identify tumor proteins displaying significant differential expression between the STS and LTS groups.
**Table S2.** Results of the SAM method to identify serum proteins displaying significant differential expression between the STS and LTS groups.
**Table S3.** Univariate Cox regression analysis for OS of the three tumor proteins of interest (AHSP, FAPB7 and TJAP1) at the mRNA level based on an analysis of the TCGA‐GB transcriptomic dataset (https://portal.gdc.cancer.gov/).
**Table S4.** Univariate Cox regression analysis for OS of the 19 serum proteins of interest at the protein level based on a serum proteome dataset for 96 IDH‐wildtype GB patients treated with a first‐line Stupp's regimen.
**Table S5.** Univariate Cox regression analysis of factors associated with PFS and OS in 93 IDH‐wildtype GB patients treated with a first‐line Stupp's regimen.

## Data Availability

The mass spectrometry proteomics data have been deposited to the ProteomeXchange Consortium via the PRIDE [83, 84] partner repository with the dataset identifier PXD045714. The clinical datasets are available from the corresponding author under the authorization of the delegation for clinical research and innovation (DRCI, CHU, Angers) and ICO (Angers).
