# Supplementary material for: Proteomics of tumor and serum samples from isocitrate dehydrogenase‐wildtype glioblastoma patients: is the detoxification of reactive oxygen species associated with shorter survival?
Source: Mol Oncol. 2024 May 27;18(11):2783–800. doi: 10.1002/1878-0261.13668 (PMC11547244; doi:10.1002/1878-0261.13668)
Supplement: Supplementary file 1 — Fig. S1. IHC staining for AHSP, FABP7 and TJAP1 on tumor tissues from STS and LTS with IDH‐wildtype GB. Table S1. Results of the SAM method to identify tumor proteins displaying significant differential expression between the STS and LTS groups. Table S2. Results of the SAM method to identify serum proteins displaying significant differential expression between the STS and LTS groups. Table S3. Univariate Cox regression analysis for OS of the three tumor proteins of interest (AHSP, FAPB7 and TJAP1) at the mRNA level based on an analysis of the TCGA‐GB transcriptomic dataset (https://portal.gdc.cancer.gov/). Table S4. Univariate Cox regression analysis for OS of the 19 serum proteins of interest at the protein level based on a serum proteome dataset for 96 IDH‐wildtype GB patients treated with a first‐line Stupp's regimen. Table S5. Univariate Cox regression analysis of factors associated with PFS and OS in 93 IDH‐wildtype GB patients treated with a first‐line Stupp's regimen. [file MOL2-18-2783-s001.zip › MOL2_13668-sup-1_Supplementary data.docx]

Table S3: Univariate Cox regression analysis for OS of the three tumor proteins of interest (AHSP, FABP7 and TJAP1) at the mRNA level based on an analysis of the TCGA-GB transcriptomic dataset (<https://portal.gdc.cancer.gov/>). * Significant difference.

Abbreviations: CI, confidence interval; GB, glioblastoma; HR, hazard ratio; OS, overall survival

| **OS** | | | | | | | | | | | | |
| --- | --- | --- | --- | --- | --- | --- | --- | --- | --- | --- | --- | --- |
|  | **Continuous variables** | | | | **Dichotomous variables_median cutoff** | | | | **Dichotomous variables_optimal cutoff** | | | |
| **Gene** |  | **HR** | **95% CI** | ***P*-value** |  | **HR** | **95% CI** | ***P*-value** |  | **HR** | **95% CI** | ***P*-value** |
| *AHSP* |  | 1.76 | [0.70-4.45] | 0.231 |  | 0.96 | [0.58-1.59] | 0.865 |  | 0.75 | [0.41-1.37] | 0.351 |
| *FABP7* |  | 1.00 | [1.00-1.00] | 0.257 |  | 1.07 | [0.64-1.78] | 0.796 |  | 0.45 | [0.21-0.95] | 0.037* |
| *TJAP1* |  | 1.03 | [0.91-1.16] | 0.651 |  | 1.01 | [0.61-1.68] | 0.956 |  | 1.96 | [0.84-4.59] | 0.119 |

Table S4: Univariate Cox regression analysis for OS of the 19 serum proteins of interest at the protein level based on a serum proteome dataset for 96 IDH-wildtype GB patients treated with a first-line Stupp's regimen. PCA on the 96 serum samples identified three outliers, which were excluded from the analysis.

* Significant difference.

Abbreviations: CI, confidence interval; GB, glioblastoma; HR, hazard ratio; PCA, principal component analysis; OS, overall survival

| **OS** | | | | | | | | | | | | |
| --- | --- | --- | --- | --- | --- | --- | --- | --- | --- | --- | --- | --- |
|  | **Continuous variables** | | | | **Dichotomous variables_median cutoff** | | | | **Dichotomous variables_optimal cutoff** | | | |
| **Protein** |  | **HR** | **95% CI** | ***P*-value** |  | **HR** | **95% CI** | ***P*-value** |  | **HR** | **95% CI** | ***P*-value** |
| AK1 |  | 1.26 | [0.95-1.68] | 0.104 |  | 1.27 | [0.83-1.94] | 0.272 |  | 2.08 | [1.29-3.34] | 0.003* |
| BPGM |  | 1.20 | [0.96-1.51] | 0.113 |  | 1.56 | [1.00-2.44] | 0.048* |  | 2.49 | [1.50-4.14] | < 0.001* |
| CA2 |  | 1.34 | [1.06-1.70] | 0.015* |  | 1.76 | [1.14-2.72] | 0.010* |  | 2.02 | [1.29-3.17] | 0.002* |
| CAT |  | 1.38 | [0.97-1.95] | 0.070 |  | 1.25 | [0.82-1.92] | 0.303 |  | 1.89 | [1.04-3.43] | 0.036* |
| EPB41 |  | 1.12 | [1.02-1.23] | 0.013* |  | 1.57 | [1.02-2.41] | 0.040* |  | 1.91 | [1.17-3.11] | 0.009* |
| FKBP1A |  | 1.17 | [0.91-1.49] | 0.224 |  | 1.24 | [0.81-1.90] | 0.326 |  | 1.85 | [1.17-2.92] | 0.009* |
| HBD |  | 1.28 | [1.01-1.62] | 0.044* |  | 1.87 | [1.21-2.88] | 0.005* |  | 2.25 | [1.41-3.57] | < 0.001* |
| MDH1 |  | 1.56 | [1.02-2.37] | 0.039* |  | 1.80 | [1.15-2.81] | 0.010* |  | 2.00 | [1.13-3.54] | 0.017* |
| MMP3 |  | 1.01 | [0.90-1.14] | 0.837 |  | 0.96 | [0.63-1.46] | 0.838 |  | 0.61 | [0.30-1.28] | 0.194 |
| PRDX1 |  | 1.32 | [0.95-1.85] | 0.101 |  | 1.41 | [0.91-2.17] | 0.121 |  | 1.49 | [0.96-2.30] | 0.074 |
| PRDX2 |  | 1.34 | [1.02-1.77] | 0.036* |  | 1.71 | [1.09-2.68] | 0.020* |  | 2.08 | [1.32-3.27] | 0.001* |
| RNH1 |  | 1.53 | [1.10-2.14] | 0.012* |  | 1.92 | [1.25-2.97] | 0.003* |  | 1.92 | [1.24-2.97] | 0.003* |
| SH3BGRL |  | 1.17 | [0.85-1.61] | 0.349 |  | 1.19 | [0.78-1.82] | 0.427 |  | 1.76 | [1.07-2.90] | 0.026* |
| SOD1 |  | 1.28 | [1.00-1.62] | 0.046* |  | 1.91 | [1.24-2.94] | 0.004* |  | 2.05 | [1.31-3.20] | 0.002* |
| STX7 |  | 1.01 | [0.92-1.11] | 0.893 |  | 0.96 | [0.63-1.46] | 0.838 |  | 0.75 | [0.47-1.19] | 0.216 |
| TXNDC17 |  | 1.16 | [0.96-1.39] | 0.118 |  | 1.43 | [0.93-2.21] | 0.103 |  | 1.72 | [1.10-2.70] | 0.017* |
| TXNL1 |  | 1.05 | [0.93-1.19] | 0.403 |  | 1.03 | [0.67-1.57] | 0.902 |  | 2.69 | [1.56-4.65] | < 0.001* |
| YWHAE |  | 1.46 | [1.05-2.03] | 0.025* |  | 1.49 | [0.97-2.30] | 0.071 |  | 2.66 | [1.19-5.97] | 0.018* |
| ZYX |  | 1.09 | [0.85-1.41] | 0.478 |  | 1.17 | [0.77-1.80] | 0.464 |  | 1.51 | [0.96-2.36] | 0.075 |

Table S5: Univariate Cox regression analysis of factors associated with PFS and OS in 93 IDH-wildtype GB patients treated with a first-line Stupp's regimen.

* Significant difference.

Abbreviations: CI, confidence interval; EOR, extent of the first resection; GB, glioblastoma; GTR, gross total resection (100%); HR, hazard ratio; KPS, Karnofsky performance score; OS, overall survival; PFS, progression-free survival; TMZ, temozolomide

|  | **PFS** | | |  | **OS** | | |
| --- | --- | --- | --- | --- | --- | --- | --- |
| **Variable** | **HR** | **95% CI** | ***P*-value** |  | **HR** | **95% CI** | ***P*-value** |
| Age (≥63 years) | 0.99 | [0.65-1.50] | 0.951 |  | 1.24 | [0.81-1.90] | 0.319 |
| Sex (female) | 0.87 | [0.56-1.37] | 0.549 |  | 0.78 | [0.49-1.24] | 0.294 |
| KPS (>80%) | 0.59 | [0.36-0.97] | 0.038* |  | 0.46 | [0.28-0.76] | 0.003* |
| Tumor laterality (left) | 0.75 | [0.50-1.15] | 0.187 |  | 0.72 | [0.47-1.10] | 0.133 |
| Tumor extent (multilobar) | 1.41 | [0.93-2.14] | 0.110 |  | 1.45 | [0.94-2.23] | 0.093 |
| EOR (GTR) | 1.05 | [0.68-1.62] | 0.835 |  | 1.05 | [0.67-1.65] | 0.822 |
| TMZ consolidation (≥6 cycles) | 0.09 | [0.05-0.17] | < 0.001* |  | 0.22 | [0.13-0.38] | < 0.001* |
| Prognostic blood score |  |  |  |  |  |  |  |
| - Score = 0 | 1 |  |  |  | 1 |  |  |
| - Score = 1 | 1.26 | [0.75-2.13] | 0.390 |  | 1.60 | [0.94-2.73] | 0.086 |
| - Score = 2 | 2.09 | [1.23-3.54] | 0.007* |  | 2.64 | [1.52-4.58] | < 0.001* |


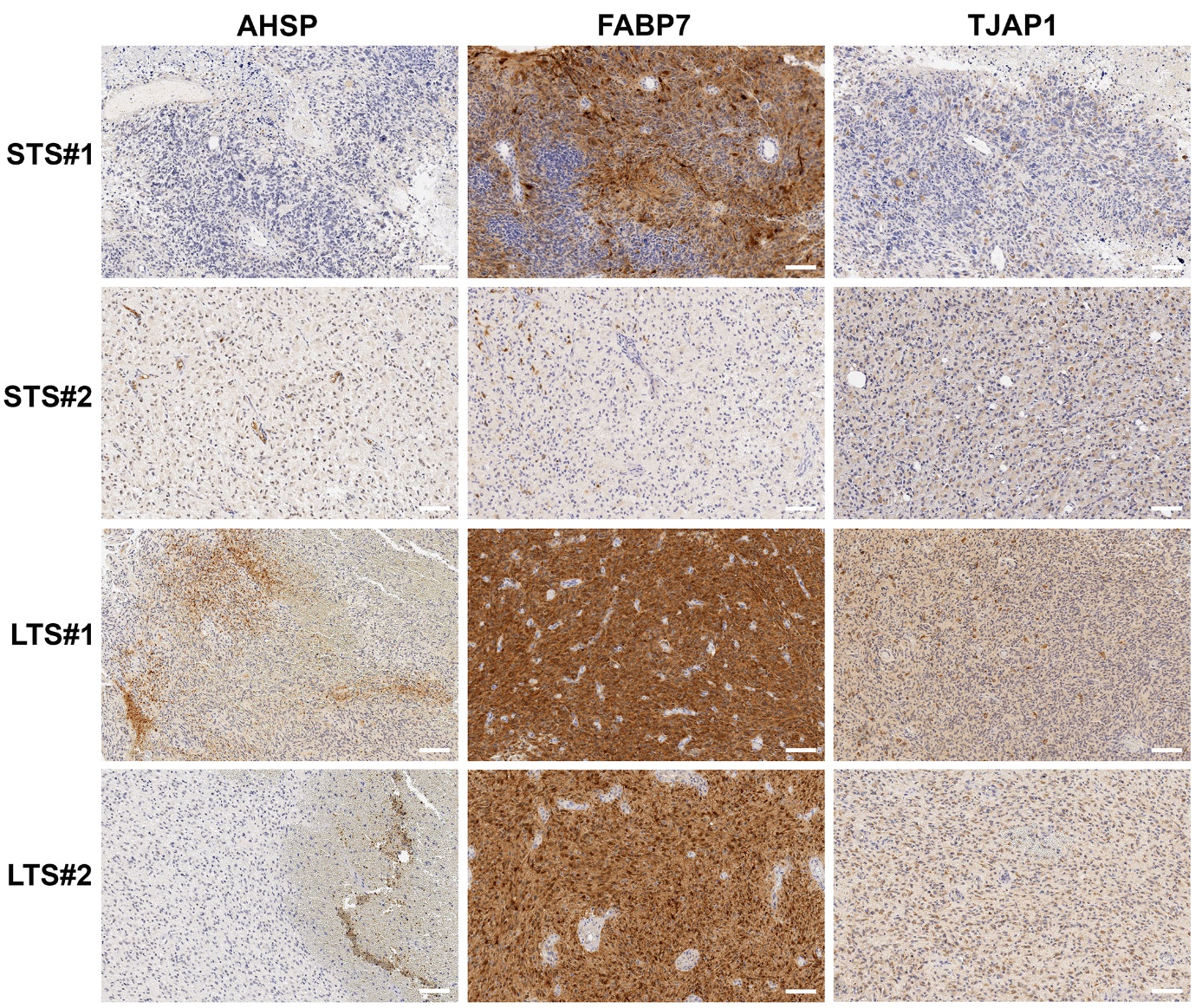


**Figure S1:** IHC staining for AHSP, FABP7 and TJAP1 on tumor tissues from STS and LTS with IDH-wildtype GB. AHSP was detected principally in blood vessels, whereas FABP7 and TJAP1 were found in the cytoplasm of tumor cells and/or in reactive glial cells. Scale bar = 100 μm.

Abbreviations: GB, glioblastoma; IHC, immunohistochemistry; LTS, long-term survivors; STS, short-term survivors
